# Development of a Biphasic-Release Multiple-Unit Pellet System with Diclofenac Sodium Using Novel Calcium Phosphate-Based Starter Pellets

**DOI:** 10.3390/pharmaceutics13060805

**Published:** 2021-05-28

**Authors:** Daniel Zakowiecki, Maja Frankiewicz, Tobias Hess, Krzysztof Cal, Maciej Gajda, Justyna Dabrowska, Bartlomiej Kubiak, Jadwiga Paszkowska, Marcela Wiater, Dagmara Hoc, Grzegorz Garbacz, Dorota Haznar-Garbacz

**Affiliations:** 1 Chemische Fabrik Budenheim KG, Rheinstrasse 27, 55257 Budenheim, Germany; Tobias.Hess@budenheim.com; 2 Department of Pharmaceutical Technology, Faculty of Pharmacy, Medical University of Gdansk, al. Gen. J. Hallera 107, 80-416 Gdansk, Poland; maja.szczepanska123@gmail.com (M.F.); kcal@wp.pl (K.C.); 3 Department of Drug Form Technology, Faculty of Pharmacy, Wroclaw Medical University, ul. Borowska 211A, 50-556 Wroclaw, Poland; Maciej.Gajda@adamed.com (M.G.); dorota.haznar@wp.pl (D.H.-G.); 4 Adamed Pharma S.A., Pienkow, ul. Mariana Adamkiewicza 6A, 05-152 Czosnow, Poland; Justyna.Dabrowska2@adamed.com (J.D.); Bartlomiej.Kubiak@adamed.com (B.K.); 5 Physiolution Polska sp. z o.o., Skarbowcow 81/7, 53-025 Wroclaw, Poland; j.paszkowska@physiolution.pl (J.P.); m.wiater@physiolution.pl (M.W.); d.hoc@physiolution.pl (D.H.); g.garbacz@physiolution.pl (G.G.); 6 Physiolution GmbH, Walther-Rathenau-Strasse 49a, 17489 Greifswald, Germany

**Keywords:** MUPS, calcium phosphate-based starter pellets, inert cores, biphasic drug release

## Abstract

Novel calcium phosphate-based starter pellets were used to develop a biphasic-release multiple-unit pellet system (MUPS) with diclofenac sodium as a model drug in the form of hard gelatin capsules. For comparative purposes, corresponding formulations based on the inert cores made of microcrystalline cellulose, sucrose and isomalt were prepared. The developed system consisted of two types of drug-layered pellets attaining different release patterns: delayed-release (enteric-coated) and extended-release. Dissolution characteristics were examined using both compendial and biorelevant methods, which reflected fed and fasting conditions. The results were collated with an equivalent commercial product but prepared with the direct pelletization technique.

## 1. Introduction

Pharmaceutical pellets, sometimes referred to as microparticulates, are free-flowing spherical particulates/beads ranging typically from 0.2–1.5 mm in size; however, other sizes are also frequently used [1,2,3]. FDA Guidance for Industry sets the maximum limit of bead sizes that should not exceed 2.5–2.8 mm [4]. The beads are used for manufacturing various multiple-unit pellet systems (MUPS), which are a kind of multiparticulate dosage form, where one or more drugs are split into numerous small independent subunits. As a drug product, MUPSs consist of drug-loaded pellets, which are either compressed into tablets or filled into hard capsules [5,6,7,8,9]. In recent years, such multiparticulate dosage forms have gained considerable popularity, and more pharmaceutical preparations of such kind are increasingly present on the market. Microparticulates are versatile drug delivery systems offering many advantages over single-unit systems and a quite high degree of flexibility in design and development of pharmaceutical dosage forms [10]. Different dosage units can be easily prepared by weighing the desired quantity of pellets without any additional formulation challenges. Microparticulates can be easily formulated as immediate-release or modified release dosage forms [11,12]. Application of special film-coating systems enables the delivery of a drug to a specific site of action within the gastrointestinal tract (GIT) [13,14]. Such multiparticulate systems are less dependent on gastric emptying, which results in lower variability in gastrointestinal transit time. They are also better distributed and less likely to cause local irritation related to higher concentrations of a drug in the same area of the GIT. Furthermore, pellets are frequently used in the preparation of controlled-release formulations. Recent research indicates that MUPSs are especially suitable for achieving controlled release with lower risk of dose-dumping [15,16]. Two or more chemically incompatible drug substances can be formulated into pellets and administered in one dosage unit [9,17,18,19]. Multiparticulates are highly beneficial for pediatric or geriatric patients with swallowing difficulties, because they enable the preparation of sprinkle capsules. The content of such capsules can be dispersed in a small amount of semisolid food or suspended in a beverage to facilitate ingestion [20,21].

Multiple-unit pellet systems are prepared by either direct pelletization or drug layering techniques. In the first case, a drug is incorporated into cores of the (drug-containing pellets); in the latter, starter pellets are coated with a drug, which forms a layer on the surface of inert cores (drug-layered pellets). Direct pelletization is normally carried out by the extrusion and spheronization of powder blend, comprising a drug substance and at least one excipient. Drug layering relies on depositing the drug particles on the outside of starter pellets (inert cores), usually with the help of a binder that is a pharmaceutically acceptable polymer. Drug layering onto inert starter pellets leads to increased surface area, better distribution of a drug, enhanced dissolution, and consequently improved bioavailability in comparison to single-unit systems (traditional tablets or capsules). Such drug-loaded pellets may be subsequently coated with different polymers in order to modify their dissolution characteristics, shield them against acidic conditions of the stomach (enteric coating), or protect from the external environment (e.g., moisture protective coating) [22,23,24,25].

The present study is an extension of previously conducted research on delayed-release formulations of diclofenac sodium (DS) [26]. This article focuses on the development and evaluation of a biphasic-release multiple-unit pellet system (MUPS) with diclofenac sodium as a model drug in the form of hard gelatin capsules. DS is available in a number of preparations for oral, intramuscular, rectal, or transdermal administration. The recommended total daily dose of 150–200 mg is normally given in divided doses of 25 mg, 50 mg or 75 mg [27,28,29]. DS is usually prepared as an enteric-coated formulation to prevent its release in the stomach, reduce gastric exposure, and consequently avoid damages to the stomach mucosa. Diclofenac sodium, with its low solubility and high permeability, is classified as a Class II drug according to the Biopharmaceutics Classification System (BCS) [30]. After oral administration, systemic absorption of diclofenac is generally rapid and directly proportional to the dose [31,32]. The need for frequent administration of DS in divided doses is due to its pharmacokinetics and may reduce patient compliance. Improving the convenience and safety of pharmacotherapy was a major driver for the development of modified-release DS formulations [33,34,35,36,37].

Diclofenac sodium pellets (DS pellets) in these studies were prepared by drug layering various types of commercial starter pellets. Many different names for starter pellets, including inert or neutral cores, spheres, beads, etc., can be found in the literature, and they will be used interchangeably in this work. There are a few types of commercial inert cores comprising different pharmaceutically acceptable material such as sucrose (sugar spheres), isomalt (isomalt pellets), and microcrystalline cellulose (MCC spheres), to name but a few. They offer different characteristics and functionality, which is well described in the literature [2,38,39,40,41]. Calcium phosphate-based starter pellets (DCPA pellets) are a novel product and a unique solution available on the market. These pellets have elevated bulk and tapped density (above 950 g/dm^3^), resulting from very high contents of anhydrous dibasic calcium phosphate (80% *w/w*). A combination of brittle calcium phosphate with 20% *w/w* of elastic material, microcrystalline cellulose, provides sufficient mechanical strength, including very low friability. Limited friability of starter pellets is desired, because it prevents the dust formation, especially at the beginning of the coating process. A summary of the product physical characteristics in comparison with other starter pellets can be found elsewhere [26,42].

The primary goal of this study was to develop and evaluate a multiple-unit biphasic-release system with diclofenac sodium. The rate of dissolution was modulated so that in vivo, after passing through the stomach, the first dose of the drug was released rapidly in duodenum in order to provide rapid relief of pain. The next dose should be released gradually to ensure effective drug concentration in the blood for a longer time and to prolong the therapeutic effect.

Reliable examination of the dissolution characteristics of MUPS that comprise subunits with different drug release modes is a quite challenging task. In the present study, release of the drug substance from the developed formulations was tested multi-directionally. The impacts of various hydrodynamic conditions were checked using modified compendial methods for DS delayed-release and extended-release tablets. Moreover, the effects of changes in pH of the environment, simulating those occurring during the gastrointestinal passage in fasted and fed states, were thoroughly tested with a reciprocating cylinder apparatus. The dissolution rate of DS from the developed formulation was compared to a commercial product of the same kind, Diclo Duo^®^ hard gelatin capsules. This preparation consists of 25 mg of diclofenac in delayed-release (DR) and 50 mg in extended-release (XR) pellets, but unlike the developed formulations, is produced with the direct pelletization technique.

## 2. Materials and Methods

Diclofenac sodium (Amoli Organics, Mumbai, India). Inert cores: calcium phosphate-based (DCPA) pellets—PharSQ^®^ Spheres CM M (Chemische Fabrik Budenheim, Budenheim, Germany), microcrystalline cellulose pellets—VIVAPUR^®^ MCC Spheres 500 (JRS Pharma, Rosenberg, Germany), sugar spheres—pharm-a-spheres^TM^ MESH 35-25 (Hanns G. Werner GmbH, Tornesch, Germany), isomalt starter pellets—galenIQ^TM^ 960 (BENEO-Palatinit GmbH, Mannheim, Germany). Film coating systems: Vivacoat^®^ FM-1M 000 (JRS Pharma, Rosenberg, Germany), Aquarius^®^ Control ENA (Ashland, Covington, KY, USA), Eudragit^®^ RL30D and Eudragit^®^ RS30D (Evonik, Darmstadt, Germany). Transparent hard gelatin capsule shells, size “00” (Pharmapol Arzneimittelvertrieb GmbH, Dägeling, Germany). DicloDuo^®^ 75 mg, modified-release capsules (Pharmaswiss Česká republika s.r.o., Praha, Czech Republic).

### 2.1. Preparation of Multiple-Unit Diclofenac Sodium Capsules

Drug loading: the starter pellets used in this study were initially calibrated between two sieves, 500 µm and 710 µm, in order to obtain grains of similar dimensions and to avoid the effect of different particle sizes on the coating process or on the analytical results. After sieving, the pellets were drug-layered with diclofenac sodium in a ProCepT 4M8-Trix Fluid-bed system (FBS) equipped with a Wurster column (ProCepT nv, Zelzate, Belgium). Around 100 g of starter pellets were coated with an aqueous suspension containing 5% *w/w* of the drug substance and 5% *w/w* of Vivacoat^®^ system, up to about 20% of weight gain. Subsequently, without breaking the process, the pellets were sprayed with purified water (intermediate coating). The use of an intermediate coating step allowed maintaining the continuity of the entire process and adjusting the process parameters before the following coating phase. Moreover, it reduced interactions between the two layers and the accumulation of static charges. The coating process settings are summarized in Table 1.

Delayed-release (DR) pellets: drug loaded pellets were coated with 20% *w/w* aqueous suspension of Aquarius^®^ Control ENA (enteric-coating) until around 10% weight gain was reached. The coating process settings are summarized in Table 1.

Extended-release (XR) pellets: drug-loaded pellets were coated with 20% *w/w* aqueous suspension containing 1:1 *w/w* mixtures of Eudragit^®^ RL30D and Eudragit^®^ RS30D up to around 6% weight gain. After completion of the coating phase, the pellets were left in a ventilated oven at a temperature of around 42 °C for 24 h (curing time). The coating process settings are summarized in Table 1.

**Figure 1:**
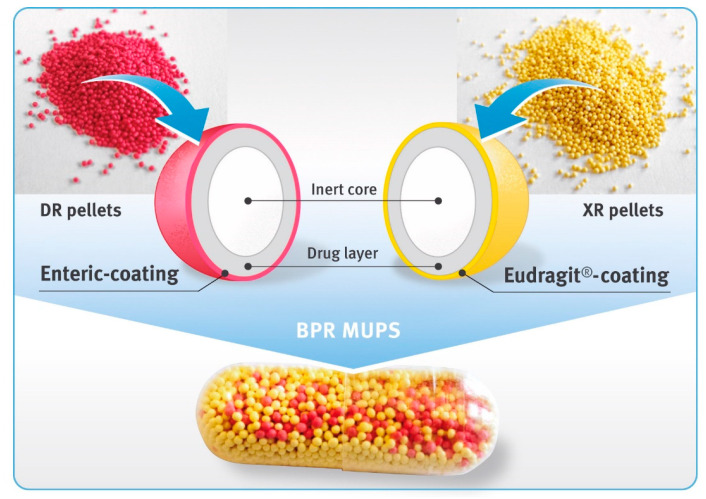
Diclofenac sodium 75 mg biphasic-release capsules comprising DR pellets (magenta beads) and XR pellets (yellow beads).

Biphasic-release multiple-unit pellet systems (BPR MUPS) in the form of hard gelatin capsules with diclofenac sodium at a dose of 75 mg were prepared by the manual filling of drug-loaded pellets into capsule shells of “00” size. An amount of DR pellets equivalent to 25 mg of diclofenac free acid, and XR pellets equivalent to 50 mg of the drug substance, were weighed, mixed and closed in transparent capsule shells (Figure 1).

Additionally, for preliminary assessment of the properties of particular types of drug-loaded pellets, hard gelatin capsules containing 25 mg of diclofenac sodium in enteric-coated pellets (DR capsules), as well as hard gelatin capsules containing 50 mg of extended-release pellets (XR capsules), were prepared and tested.

### 2.2. Characterization of Starter and Drug-Layered Pellets

The bulk density of pellets before and after coating was assessed by measurement of the unsettled apparent volume of 200 g samples in a graduated 250 mL cylinder, as described in USP/NF Chapter <616>, Method I (measurement in a graduated cylinder).

Shape factor analysis for the pellets was carried out using a digital microscope Keyence^®^ VHX-5000 (magnification of 50×) equipped with image analysis software (Keyence Corporation, Osaka, Japan). Roundness (degree of circularity) and convexity (envelope curve) were calculated based on Equations (1) and (2), respectively. The results are based on measurements of around 700 pellets.

roundness = 4πA/P^2^, (1)

convexity = Pc/P,(2)

where A is the projected two-dimensional area of a particle, P is the perimeter of a particle, and Pc is the convex envelope perimeter.

Drug content was determined spectrophotometrically at a detection wavelength of 276 nm using a T70 UV/VIS Split-Beam Spectrophotometer (PharmaTest AG, Hainburg, Germany) equipped with flow-through quartz cuvettes with 10 mm optical path length. Sample solutions nominally containing around 0.01 mg/mL of diclofenac sodium were prepared by sonication of a suitable quantity of pellets in a mixture of methanol with 0.05 M phosphate buffer pH 7.5 (1:1 *w/w*), and subsequent dilution with the same buffer solution to the desired concentration of the analyte. The results were calculated in reference to the calibration curve of the reference material (diclofenac sodium) dissolved in 0.05 M phosphate buffer pH 7.5.

### 2.3. Scanning Electron Microscopy and Raman Imaging

Scanning electron microscopy (SEM) and Raman microscopy (RM) were used in the analysis of DR and XR pellet cross-sections in order to evaluate the thickness and quality of the coating layers. Prior to the analysis, the pellets were embedded in an epoxy resin on a sample holder and cut with the help of a laboratory ultramicrotome EM TRIM2 (Leica Microsystems, Wetzlar, Germany). SEM analysis was carried out with an EVO 25LS scanning electron microscope (Carl Zeiss Microscopy GmbH, Oberkochen, Germany). SEM micrographs were recorded at a magnification of 500× with an acceleration voltage of 25 kV under extended vacuum pressure mode. 

Raman imaging was carried out using an Alpha300R confocal Raman microscope (WITec GmbH, Ulm, Germany). A 785 nm laser was used in combination with Zeiss 50× LD NA 0.55 objective and 300 g/mm, BLZ = 750 nm grating. Raman images were collected using two spatial resolutions, one lower than 10 µm and one higher than 3 µm.

### 2.4. Dissolution Tests (Modified Compendial Methods)

At the beginning, the dissolution characteristics of DR and XR pellets were tested separately, following the USP42/NF37 monographs for diclofenac sodium delayed-release tablets and diclofenac sodium extended-release tablets, respectively.

Enteric-coated pellets: the test consisted of two stages (acidic and buffer), and the tested samples were transferred from one vessel to another between these stages. In the case of tablets, a paddle apparatus was applicable because transfer of the tablets was not a major problem. In the case of enteric-coated pellets, however, basket apparatus was more convenient for use. Thus, capsules containing 25 mg of diclofenac sodium in DR pellets were placed in basket apparatus (USP apparatus 1), and the dissolution test was carried out according to the monograph for diclofenac sodium delayed-release tablets given in USP42/NF37 using a rotational speed of 100 rpm. The use of the same procedure for the analysis of delayed-release diclofenac sodium formulations was reported elsewhere [26].

Extended-release pellets: capsules containing 50 mg of diclofenac sodium in XR pellets were examined under two conditions, as described in the monograph for diclofenac sodium extended-release tablets in USP42/NF37 (Test 2 and 3). According to Test 2, the capsules were placed in a paddle apparatus (USP apparatus 2, rotational speed of 50 rpm), and the analysis was carried out for 10 h. To prevent the capsules from floating to the surface, they were placed in the sinkers prior to being immersed in the dissolution liquid. According to Test 3, the capsules were tested using a basket apparatus (USP apparatus 1, rotational speed of 100 rpm) within 16 h. Thus, the effect of different hydrodynamic conditions on the dissolution rate of diclofenac sodium was evaluated.

Biphasic-release diclofenac sodium capsules (BPR MUPS): there is no compendial method for such a product. The tests were carried out for 24 h in two stages. In the first, acidic phase, the BPR MUPS capsules were tested according to the monograph for diclofenac sodium delayed-release tablets given in USP42/NF37, using basket apparatus (USP apparatus 1) and a rotational speed of 100 rpm. After the acid stage (120 min maceration in 0.1 M HCl), the tested samples were transferred to 0.05 M phosphate buffer pH 7.5 and the test was continued for 22 h following the USP Dissolution Test 2, as described in the monograph for diclofenac sodium extended-release tablets in USP42/NF37, but using the baskets and a rotational speed of 100 rpm.

All analyses were carried out in a dissolution apparatus PTWS 820D (Pharma Test Apparatebau AG, Hainburg, Germany), and collected samples were analyzed offline using a UV/Vis spectrophotometer T70 (PG Instruments Ltd., Leicestershire, UK) at a wavelength of 276 nm (optical path length of 10 mm).

### 2.5. Dissolution under Conditions Simulating pH Changes in Fasted and Fed States

Biphasic-release diclofenac sodium capsules and the commercial products were tested in variable media that simulated pH changes in fasted and fed states. Tests were performed using USP Apparatus 3—reciprocating cylinder (Bio-Dis, Agilent Technologies Inc., Santa Clara, CA, USA) with an automatic sampling system (850-DS, Agilent Technologies Inc., Palo Alto, CA, USA). Dissolution was carried in 250 mL vessels equipped with inner tubes with bottoms consisting of a 100 mesh (150 micron) polypropylene screen. Examined capsules were placed on the sieve of the inner tubes and agitated at 8, 10 or 15 dips per minute (dpm) and 10 cm stroke length for 22 or 24 h in the following sequences:

(A) Simulation of fasted state:

- 120 min in 0.1 M hydrochloric acid pH 1.0 at 15 dpm;
- 20 min in 0.05 M phosphate buffer solution pH 5.6 at 15 dpm;
- 100 min in 0.05 M phosphate buffer solution pH 6.8 at 15 dpm;
- 1080 min in 0.05 M phosphate buffer solution pH 6.8 at 10 dpm;

(B) Simulation of fed state:

- 30 min in 0.05 M phosphate buffer solution pH 4.5 at 8 dpm;
- 60 min in 0.05 M phosphate buffer solution pH 3.5 at 8 dpm;
- 150 min in 0.01 M hydrochloric acid pH 2.0 at 15 dpm;
- 30 min in 0.05 M phosphate buffer solution pH 5.6 at 15 dpm;
- 330 min in 0.05 M phosphate buffer solution pH 6.8 at 15 dpm;
- 840 min in 0.05 M phosphate buffer solution pH 6.8 at 10 dpm.

For sample preparation, 5 mL of dissolution medium were taken (without refilling) and filtered through a 0.2 µm syringe filter (Minisart^®^, Sartorius Stedim Poland sp. z o.o., Kostrzyn Wlkp., Poland). Then, 1 mL of the sample was diluted with 3 mL of 0.05 M phosphate buffer solution pH 6.8 and analyzed using a UV–Vis spectrophotometer Jasco V-650 (ABL&E-JASCO Polska Sp. z o.o., Cracow, Poland) at wavelength λ = 276 nm.

## 3. Results

### 3.1. Characterization of Starter and Drug-Layered Pellets

**Figure 2:**
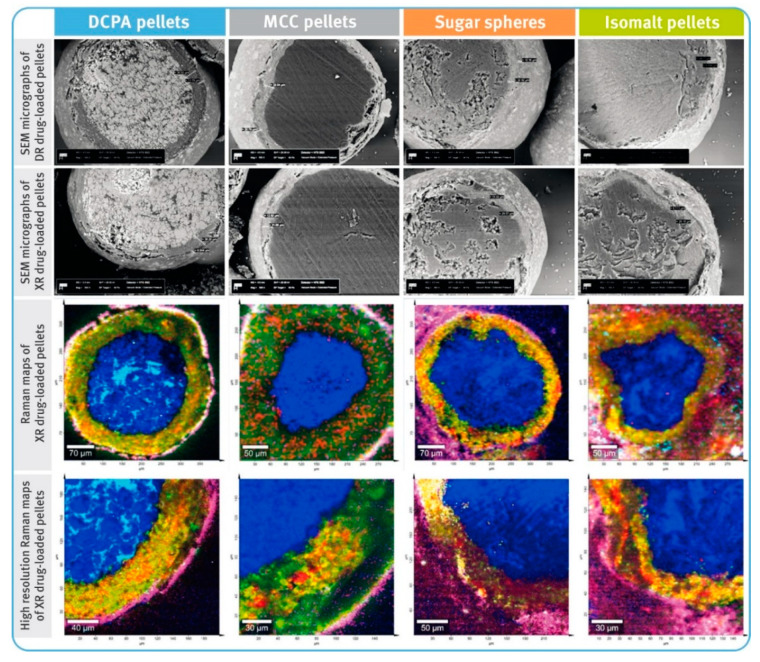
SEM micrographs of cross-sections of diclofenac sodium DR and XR pellets (magnification of 500×) and Raman maps of XR pellets (spatial resolution of 10 µm and high resolution of 3 µm); location of diclofenac sodium marked with red color, core material with different shades of blue, hypromellose—green to yellow, and polymethacrylate-based coating—pink.

**Figure 3:**
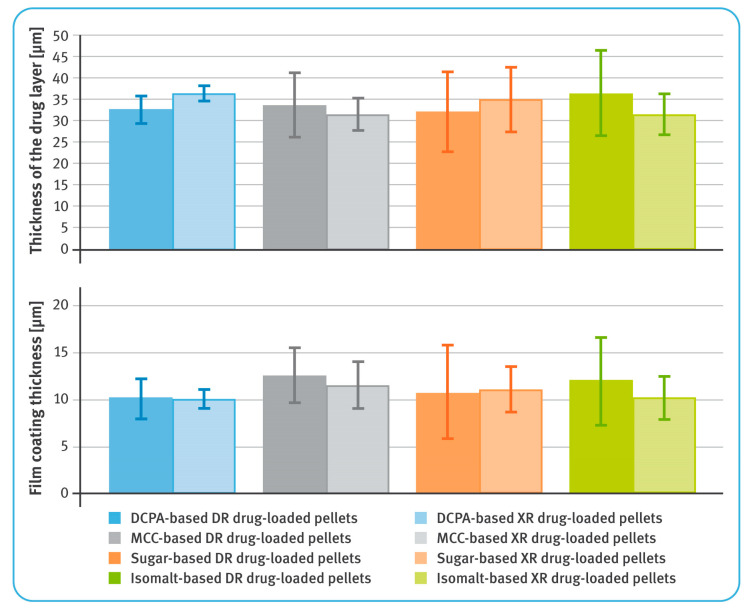
Thicknesses of layers containing diclofenac sodium (upper) and film coatings (lower) (mean of *n* = 6, SD are indicated by the error bars).

Table 2, Table 3 and Table 4 and Figure 2 and Figure 3 show comparisons of selected properties of drug-loaded diclofenac sodium pellets. The highest bulk density was observed for the pellets based on DCPA, and the lowest for isomalt pellets (Table 2). Both DR and XR coatings had no significant impact on the density of the microparticulates. Similarly, values of shape factors (roundness and convexity) of both coated and uncoated pellets were found to be the highest for DCPA and the lowest for isomalt pellets (Table 3), with rather little effect of the film coatings. 

In general, the average thicknesses of the coating layers (both containing the drug substance as well as dissolution-controlling layers) were very similar for all pellets, regardless of the chemical nature of their cores (Figure 2). For both DR and XR pellets, thicknesses of the layers with diclofenac sodium were in the range of 31–37 µm. For DR pellets, the average thicknesses of the gastro-resistant layers were between 10 and 13 µm, and for XR pellets, the thicknesses of dissolution-controlling layers were around 10–12 µm. It was observed that in the case of less spherical and less convex pellets, the layers were unevenly distributed (Figure 2). The average content of diclofenac sodium in the drug-loaded pellets was similar for all tested multiparticulates and amounted to around 7.2–7.6% *w/w* of DR pellets, and 7.9–8.2% *w/w* of XR pellets (Table 4).

### 3.2. Dissolution Testing of Diclofenac Sodium DR Pellets

**Figure 4:**
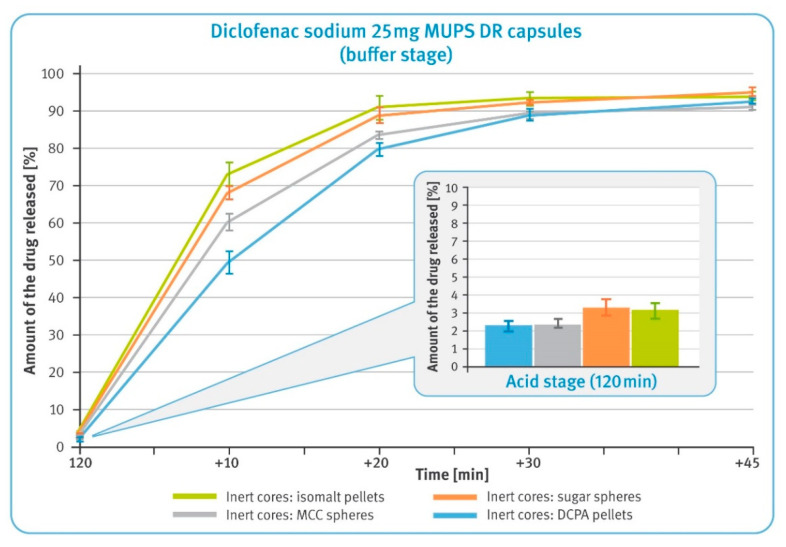
The release of the drug substance from diclofenac sodium 25 mg DR hard gelatin capsules: 2 h incubation in 0.1 M HCl (in the smaller box) and in the buffer phase (phosphate buffer solution, pH 6.8) (mean of *n* = 6, SD is indicated by the error bars).

**Figure 5:**
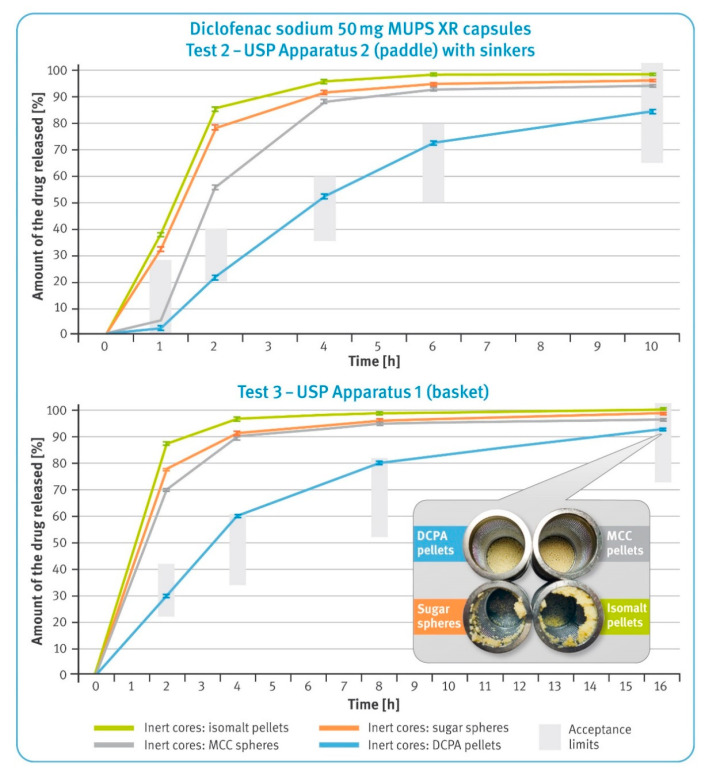
Dissolution rate of the drug substance from diclofenac sodium 50 mg XR hard gelatin capsules in 0.05 M phosphate buffer, pH 7.5 following USP Test 2 (upper) and USP Test 3 (lower) (mean of *n* = 6, SD is indicated by the error bars). In the smaller box: residues of diclofenac sodium XR capsules in baskets after completing Test 3.

The release of the drug substance from capsules containing enteric-coated pellets in amounts equivalent to 25 mg of diclofenac sodium was first examined after 2 h maceration in 0.1 M hydrochloric acid and neutralization of the solution with 5 M sodium hydroxide. The results are shown in a smaller box in Figure 4, and for all pellet types, they were within the limits required for this type of preparations, i.e., not more than 10% of the labeled content should be dissolved. The amount of diclofenac sodium dissolved was lower in the case of DCPA- and MCC-based pellets by about 1% compared to those comprising sucrose or isomalt. It should be noted that the latter two types of pellets showed a tendency to float and gather in the upper part of baskets. An example of similar behavior is shown in the smaller box in Figure 5.

In the buffer phase, the dissolution of diclofenac from all types of pellets is rapid and meets pharmacopeial requirements of not less than 80% being released within the first 45 min (Figure 4). However, it can be observed that for the first 30 min, the dissolution rate was slower for microparticulates with water-insoluble particles, i.e., DCPA and MCC, and faster for water soluble particles, i.e., isomalt and sugar.

### 3.3. Dissolution Test of Diclofenac Sodium XR Pellets

The dissolution results of the capsules containing extended-release pellets with 50 mg of diclofenac sodium are shown in Figure 5. The upper graph shows the dissolution profiles obtained in the paddle apparatus (Test 2), while the lower graph shows the release profiles recorded in basket apparatus (Test 3). In both graphs, the green bars indicate the requirements as per USP42/NF37 monographs for diclofenac sodium delayed-release tablets.

In both cases, very rapid release of the drug substance from the pellets with water-soluble cores was observed. In the case of pellets with DCPA cores, the control of the release process was the most effective. Moreover, this type of microparticulates met requirements for both Tests 2 and 3, as per the pharmacopeial monograph. The microparticulates containing water-soluble cores showed a tendency for flotation (the smaller box in Figure 5), which was also observed for DR pellets.

### 3.4. Dissolution Test of Diclofenac Sodium 75 mg BPR Hard Gelatin Capsules (Modified Compendial Methods)

**Figure 6:**
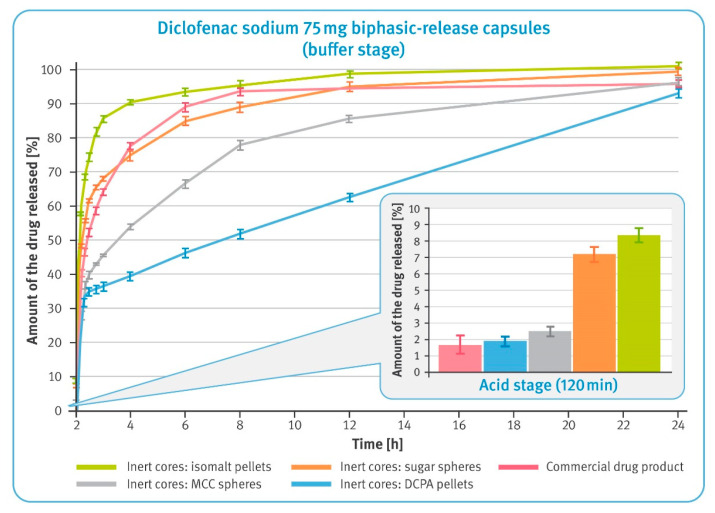
The release of the drug substance from diclofenac sodium 75 mg biphasic-released hard gelatin capsules in the acid phase, after 2 h incubation in 0.1 M HCl (in the smaller box) and in the buffer phase (phosphate buffer solution, pH 7.5) (mean of *n* = 6, SD is indicated by the error bars).

Dissolution of the drug substance from BPR hard gelatin capsules containing 25 mg of diclofenac sodium in DR pellets and 50 mg in XR pellets was first examined after 2 h maceration in 0.1 M hydrochloric acid and neutralization of the solution with 5 M sodium hydroxide. The results in the smaller box in Figure 6 show that all tested preparations met the requirements for enteric-coated formulations, i.e., not more than 10% of the labeled content should be dissolved under acidic conditions. It may be noted, however, that the amount of diclofenac sodium dissolved in the two-hour acid stage was the lowest for the commercial product and the pellets with water-insoluble cores. Microparticulates with isomalt- and sugar-based cores released a significantly higher amount of the drug substance. In a 22 h buffer stage, the dissolution rates from these pellets were much higher when compared with pellets based on water-insoluble cores. Similarly, rapid dissolution was observed for the commercial product. Microparticulates with water-insoluble cores showed a much better controlled release rate. It should be noted that in the buffer stage, the release of diclofenac sodium from DCPA-based pellets was almost linear until the end of this phase.

### 3.5. Dissolution under Conditions Simulating pH Changes in Fasted and Fed States

**Figure 7:**
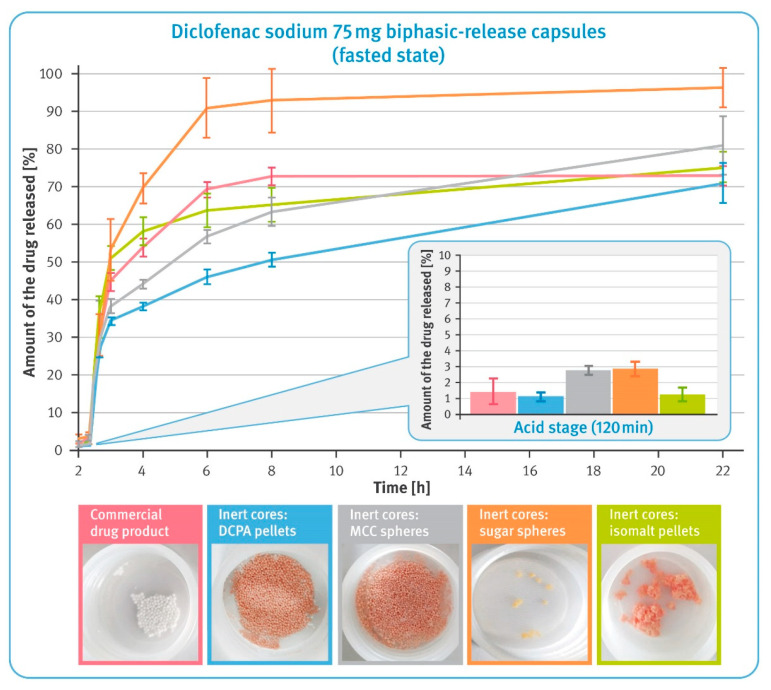
The release of the drug substance from diclofenac sodium 75 mg biphasic-release hard gelatin capsules under conditions simulating pH changes in fasted state in USP apparatus 3 (mean of *n* = 3, SD is indicated by the error bars). Underneath are residues of diclofenac sodium BPR capsules in tubes after completing the test.

The dissolution behavior of BPR capsules in a small volume of fluid was studied using reciprocating cylinder apparatus. Tests simulating pH changes in the fasted state started with a 2 h maceration in hydrochloric acid, and the results are shown in a smaller box in Figure 7. It was observed that in this phase, all examined formulations released very small amounts of diclofenac sodium, which did not exceed 3% of the labelled content. As the pH of the dissolution fluid increased, rapid release of the drug substance from the multiparticulates began. It was much faster in the case of pellets with water-soluble cores compared to those with insoluble cores. Furthermore, it was observed that for isomalt pellets and the commercial product, the initial rapid release was followed by significant slowing down of this process after 6 h of the test. Multiparticulates containing MCC- and DCPA-based cores showed a very steady dissolution rate, which in the latter case was almost linear. Upon completing the test, the residues of the tested formulations were found in the tubes, as shown in the pictures of Figure 7. It was noted that in the case of pellets with water-soluble cores and the commercial product, a significantly lower amount remained after the 22 h test. For pellets based on the insoluble material, virtually all of the material remained after testing. The sugar-based pellets were almost completely dissolved, and the isomalt pellets clumped together to form large lumps.

**Figure 8:**
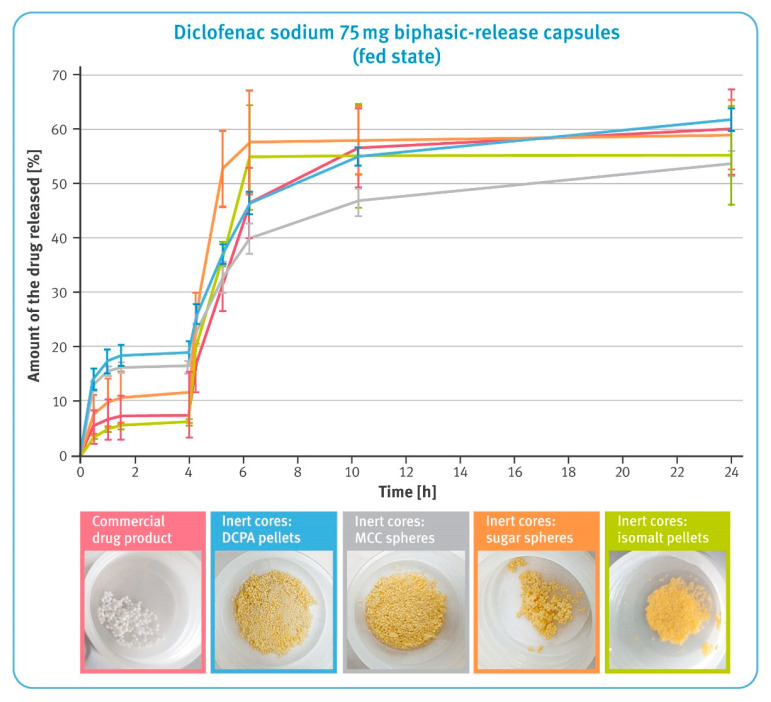
The release of the drug substance from diclofenac sodium 75 mg biphasic-release hard gelatin capsules under conditions simulating pH changes in the fed state in USP apparatus 3 (mean of *n* = 3, SD indicated by the error bars). Underneath are residues of diclofenac sodium BPR capsules in tubes after completing the test.

Results of dissolution under conditions simulating pH changes in the fed state are shown in Figure 8. During the first 4 h, the release of diclofenac sodium from pellets comprising water-soluble cores and the commercial product was significantly lower compared to MCC- and DCPA-based microparticulates. The subsequent increase in pH of the dissolution fluid caused a rapid release of the drug substance from the all examined preparations; however, the speed of this process was the highest for isomalt- and sugar-based pellets, and the slowest for MCC-based pellets. Interestingly, the release of the drug substance from the pellets with water-soluble cores practically stopped after 6 h of the test, while gradual dissolution was still observed for the other pellets. Pictures under the graph in Figure 8 show the residues in the tubes after completing the test. After a 24 h dissolution, a smaller quantity of pellets with water-soluble cores as well as the commercial product remained compared to the microparticulates based on an insoluble material, where virtually all material remained after testing. The sugar- and isomalt-based pellets showed a tendency to stick together and form lumps.

## 4. Discussion

In the present study, biphasic-release multiple-unit pellet systems (BPR MUPS) with diclofenac sodium as a model drug in the form of hard gelatin capsules were developed and evaluated. This was achieved by placing two types of multiparticulates into one capsule shell, as shown in Figure 1. The first type of the multiparticulates constituted the DR pellets. They had an enteric coating to prevent the release of the drug substance in a low pH environment (typical for the fasting stomach), and allow the entire amount of the drug substance to dissolve in the higher pH, prevailing in the distal parts of the GIT. This approach, on the one hand, avoids local irritation of the stomach, and on the other hand, reduces the likelihood of the formation of diclofenac free acid, which is very poorly soluble in aqueous media of both acidic and neutral pH [43]. The second type of multiparticulates used in BPR MUPS were XR pellets that were coated with a polymer, prolonging the release of the drug for a longer time. Their function was to maintain the dissolution of the drug over an extended period of time, optimally at a constant rate. The combination of the delayed- and extended-release formulation allowed preparation of a once-a-day dosage form, both with a rapid onset of pain relief and prolonged treatment of this pain. From the perspective of patients suffering from chronic diseases, the development of such medicines that could be taken less frequently, although still ensuring maintenance of the therapeutic concentration in the body for a long time, would be highly beneficial. It could also enhance patient compliance as well as the safety of pharmacotherapy [44,45].

Both DR and XR multiparticulates were prepared by the drug layering of four types of commercial starter pellets. Two of them, sugar spheres and isomalt pellets, showed good solubility in aqueous media. The others, MCC spheres and DCPA pellets, were insoluble in water. All inert cores used in this study were initially normalized between two sieves, 500 µm and 710 µm, in order to obtain grains of similar dimensions and to avoid the effect of different particle sizes on the quality of coatings, and furthermore, on the results of the analyses. However, the pellets differed in terms of bulk density, sphericity and convexity. Convexity is a measurement of particle edge roughness and seems to be correlated with pellet density (see Table 2 and Table 3). It should be mentioned that in the case of sugar and isomalt pellets, which have a lower bulk density (and thus occupy a greater volume), filling the capsules was quite challenging.

The prerequisite for drug-containing layers was achieved, and for all types of the starting pellets the average thicknesses of these layers were very similar, ranging from 31 to 37 µm (see Figure 3), despite differences in the density and shape factors of the inert cores. Close examination of bead cross-sections by SEM and RM techniques revealed differences in the layer thickness within a given type of pellets (Figure 2). These differences were manifested in the standard deviation values and correlated with sphericity and convexity of neutral cores. Thus, the lowest value of the standard deviation was noticed for DCPA-based pellets and the largest for sugar- and isomalt-based pellets (see Table 3). Nevertheless, all these variables had no significant effect on the content of the drug substance, which was very similar, averaging 7.2–7.6% *w/w* for DR pellets and 7.9–8.2% *w/w* for XR pellets, with relatively low standard deviation values (see Table 4).

The quality of the functional coatings of both DR and XR pellets was assessed in terms of their thickness and uniformity of the core coverage, as well as by evaluating their effectiveness in the performance tests. The average thicknesses of the gastro-resistant layers ranged from 10 to 13 µm, and those of the dissolution-controlling layers from 10 to 12 µm. Depending on the type of the inert core, the layer thickness showed more or less variation. As the sphericity and convexity improved, the standard deviation values decreased. Thus, the smallest variation in enteric-coating thickness was observed for DCPA-based pellets, and the highest for isomalt pellets (see Figure 3 and Table 3).

In the performance test, all examined DR multiparticulates demonstrated their gastro-resistance and released much less than 10% of the labeled amount of diclofenac sodium within 2 h of the test conducted in 0.1 M hydrochloric acid (see the smaller box in Figure 4). In the case of pellets containing water-soluble cores, a slightly higher release of the drug substance could be detected. In the buffer stage, all tested formulations demonstrated rapid dissolution and released the whole amount of the drug within the required 45 min (see Figure 4). However, it can be noted that the rate of dissolution varied among tested formulations, being faster for very soluble isomalt-based pellets and slower for insoluble DCPA-based pellets. Considering that the average thicknesses of all layers were identical for all pellets, this phenomenon must be related to the chemical nature of the inert cores and their interaction with the dissolution medium. 

Very similar observations were made when studying the release of diclofenac sodium from the XR pellets. Despite using the same dissolution-controlling coating system of identical thickness in all formulations, the pellets with water-soluble cores released the drug substance very rapidly in both types of dissolution apparatus (see Figure 5). The observed flotation of these pellets (see the smaller box of Figure 5) was probably due to the fact that during the long-lasting test, the materials contained in the cores dissolved, and the resultant solutions of relatively high concentrations diffused through the wetted polymer layers. In addition, concentrated solutions of sucrose or isomalt formed inside the pellets, generating a high osmotic pressure, which could facilitate the dissolution of diclofenac sodium. Such effects of the osmotic pressure built up inside the pellets and film-coating thickness on the rate of drug release have been described elsewhere [46,47]. It can be assumed that the thicker release-controlling layer or the use of polymers with a higher density would result in a slower release of the drug substance.

Interestingly, even though MCC cores do not dissolve in an aqueous media, multiparticulates based on them showed a fairly rapid release of diclofenac sodium. In addition, a significant effect of different hydrodynamic conditions on the release from cellulose-based pellets was also observed. In the basket apparatus, the release rate during the first hours of the dissolution test was higher than in the paddle apparatus. Presumably, the swelling of MCC-based pellets in contact with water (reported elsewhere) may play a role here. Increasing the volume of inert cores can cause the release-controlling layer to crack or decrease in thickness, resulting in faster release of the drug substance [48,49].

The calcium phosphate pellets showed stable, controlled release of the drug substance under various hydrodynamic conditions. When comparing the results obtained in both basket and paddle apparatus with the pharmacopoeial requirements for each assay, Tests 2 and 3, only DCPA-based multiparticulates met the required limits (see the upper and lower graphs in Figure 5).

BPR MUPS developed in this study consisted of both DR and XR pellets placed in a capsule shell. In a series of dissolution tests, the performance of the developed formulations was examined and compared with the commercial product of the same type but made from pellets produced by direct pelletization.

The first test, carried out with modified compendial methods, was conducted in basket apparatus and consisted of two stages. In the first, acid stage, all formulations demonstrated to be satisfactory gastro-resistance and released less than 10% of the labeled amount of diclofenac sodium within 2 h of the test conducted in 0.1 M hydrochloric acid (see the smaller box in Figure 6). It was observed that multiparticulates containing water-soluble cores released significantly more of the drug substance than the other formulations tested. This must have been due to the high solubility of these cores and the osmotic pressure built up inside the pellets. Considering the low release results in the acid phase of DR pellets (see Figure 4), this should have been related to the higher dissolution rate of diclofenac sodium observed in the case of XR pellets (see Figure 5).

In the second phase of the test, a clear correlation between the solubility of inert cores and release rate was observed (comparisons of solubility of various starter pellets can be found elsewhere [44]). Freely soluble isomalt-based pellets showed a rapid release of diclofenac sodium; sugar pellets containing 80% of sucrose were much slower. Interestingly, dissolution of the drug substance from the commercial product was found to be as fast as those from sugar-based multiparticulates. Much better control of the release rate was observed for pellets with water-insoluble inert cores. Notably, in the case of DCPA-based pellets, it was observed that the release of the drug substance occurred at a precisely controlled rate corresponding to zero-order kinetics. The dissolution rate from MCC-based multiparticulates was faster, which might be related to the assumed swelling of their cores and the effect of this phenomenon on the polymer layer.

The dissolution behavior of BPR capsules and the commercial product under conditions simulating pH changes in the fasted and fed state was tested using reciprocating cylinder apparatus. USP Apparatus 3 provided much stronger agitation and a smaller volume of dissolution fluid compared to the paddle or basket, which further reflected some of the conditions affecting the drug during the gastrointestinal passage. In this environment, incomplete dissolution of diclofenac sodium was observed during the 22–24 h tests. In addition to the reduced amount of dissolution liquid, two phenomena played a significant role in the case of the water-soluble pellets. On the one hand, the dissolution of the inert cores was accompanied by flotation of the pellets. In some cases, smaller beads escaped from the inner tubes through larger openings of the polypropylene screens, which resulted in the higher variability of the results. On the other hand, the pellets tended to stick together and form large agglomerates, which limited contact of the pellets with the dissolution liquid (see the photos below the graphs in Figure 7 and Figure 8).

In the acid phase of the test simulating fasting conditions, all formulations confirmed the effectiveness of the enteric coating and released less than 3% of the labeled content of diclofenac sodium (see the smaller box in Figure 7). The effect of the core characteristics became apparent in higher pH environments. As in earlier tests, a higher release rate was evident for pellets based on water-soluble cores, as well as for the commercial product. An increase in the pH of the dissolution liquids was accompanied by a rapid release of diclofenac sodium from all tested formulations. Although this process practically stopped after 6 h of testing for some of the multiparticulates, for reasons described earlier, the effect of core solubility on the dissolution rate of the drug is clearly visible (see Figure 7). Pellets based on MCC and DCPA showed very controlled dissolution, and the process had almost linear characteristics for these pellets. In comparison, the pellets containing sugar cores released almost all of the active substance content and this was due to their practically complete dissolution (see the photos below the graphs in Figure 7).

An interesting situation was observed in conditions simulating pH changes in the fed state, when the pH of the stomach is usually slightly higher. In the first phase of the study, pellets based on water-insoluble cores released significantly more diclofenac sodium than the other formulations tested (see Figure 8). Given the previous test results, it is reasonable to assume that it would be more beneficial to take such preparations on an empty stomach. The further part of the test showed the favorable dissolution profile of the commercial product. The DCPA-based pellets released diclofenac sodium with an identical rate; however, when comparing the results of their release in conditions simulating fasted and fed states, a lower sensitivity to the prevailing conditions could be observed. In contrast, MCC-based pellets showed a much lower release rate under conditions simulating the fed state.

## 5. Conclusions

The aim of this study was to develop multi-unit biphasic release systems (BPR MUPS) for diclofenac sodium using different types of neutral starter pellets. A series of performance tests demonstrated that control of the dissolution of the drug substance from the developed formulations was clearly related to the water solubility of their inert cores. It was observed that the water-insoluble starter pellets (MCC spheres and DCPA-based pellets) were able to control the rate of release much better than soluble sugar spheres or isomalt pellets. In the case of the latter, it can be assumed that the use of more efficient polymers or a greater amount of them would result in a decrease in the dissolution rate. In this respect, MCC and DCPA starter pellets offer a very attractive opportunity of improving productivity and controlling the dissolution rate of drugs with a smaller quantity of polymers. Calcium phosphate-based pellets appear to be a particularly promising prospect.

## Figures and Tables

**Table 1:** Summary of process parameters used during pellet coating.

Process Parameter	Drug Loading	Enteric Release Coating	Functional Film Coating
Inlet airflow rate	0.31 ± 0.1 m^3^/min	0.32 ± 0.1 m^3^/min	0.30 ± 0.1 m^3^/min
Inlet air temperature	57 ± 2 °C	56 ± 2 °C	31 ± 2 °C
Product temperature	40 ± 2 °C	41 ± 2 °C	26 ± 2 °C
Spraying pressure	1.1 ± 0.2 bar	1.1 ± 0.2 bar	1.0 ± 0.2 bar
Coating mixture flow rate	1.3 g/min	1.0 g/min	1.0 g/min
Drying time	10 min	30 min	30 min
Curing time and temperature	-	-	24 h at 42 ± 2 °C

**Table 2:** Comparison of the bulk density of starter and drug-loaded pellets (mean of 3 independent determinations).

Inert Cores	DCPA Pellets	MCC Spheres	Sugar Spheres	Isomalt Pellets
Starter pellets	1.06 ± 0.02 g/mL	0.89 ± 0.01 g/mL	0.87 ± 0.01 g/mL	0.81 ± 0.02 g/mL
DR drug-loaded pellets	1.02 ± 0.01 g/mL	0.85 ± 0.02 g/mL	0.88 ± 0.01 g/mL	0.86 ± 0.02 g/mL
XR drug-loaded pellets	1.05 ± 0.02 g/mL	0.92 ± 0.02 g/mL	0.89 ± 0.02 g/mL	0.90 ± 0.02 g/mL

**Table 3:** Comparison of the roundness and convexity of starter and drug-loaded pellets.

Inert Cores	DCPA Pellets		MCC Spheres		Sugar Spheres		Isomalt Pellets	
Parameter	Roundness	Convexity	Roundness	Convexity	Roundness	Convexity	Roundness	Convexity
Starter pellets	0.891 ± 0.015	0.995 ± 0.004	0.874 ± 0.023	0.993 ± 0.007	0.845 ± 0.030	0.981 ± 0.011	0.787 ± 0.042	0.968 ± 0.013
DR drug-loaded pellets	0.892 ± 0.017	0.998 ± 0.005	0.869 ± 0.028	0.994 ± 0.008	0.845 ± 0.057	0.978 ± 0.027	0.785 ± 0.082	0.956 ± 0.047
XR drug-loaded pellets	0.890 ± 0.022	0.993 ± 0.010	0.873 ± 0.026	0.991 ± 0.012	0.867 ± 0.034	0.991 ± 0.014	0.828 ± 0.034	0.985 ± 0.010

**Table 4:** Content of diclofenac sodium in drug-loaded pellets (mean of 3 independent determinations).

Inert Cores	DCPA Pellets	MCC Spheres	Sugar Spheres	Isomalt Pellets
DR drug-loaded pellets	7.37 ± 0.01%	7.62 ± 0.04%	7.31 ± 0.07%	7.24 ± 0.05%
XR drug-loaded pellets	8.02 ± 0.06%	8.04 ± 0.08%	7.93 ± 0.01%	8.18 ± 0.09%

## Data Availability

All data/results collected in this study are presented in the article.
